# Occurrence of Tick‐Borne Pathogens in *Rhipicephalus sanguineus* Sensu Lato From Domestic Dogs in Kumasi, Ghana

**DOI:** 10.1155/vmi/8881048

**Published:** 2026-01-28

**Authors:** Sandra Abankwa Kwarteng, Jubin Osei Mensah, Patrick Kwasi Obuam, Enoch Ago Odenteh, Priscilla Denkyira Foriwaah, Anne Ifunanya Mbelede, Edwin Dziwornu, Ewurabena Oduma Duker, Jessica Dufie Boakye, Gayheart Deladem Agbotse, Jennifer Nyamekye Yanney, Millie-Cindy Aba Aude Koffi, Michael E. DeWitt, Seth Offei Addo

**Affiliations:** ^1^ Department of Theoretical and Applied Biology, College of Science, KNUST, Kumasi, Ghana, knust.edu.gh; ^2^ School of Veterinary Medicine, Kwame Nkrumah University of Science and Technology, Kumasi, Ghana, knust.edu.gh; ^3^ School of Public Health, Kwame Nkrumah University of Science and Technology, Kumasi, Ghana, knust.edu.gh; ^4^ Virology Department, Noguchi Memorial Institute for Medical Research, University of Ghana, Legon, Accra, Ghana, ug.edu.gh; ^5^ Parasitology Department, Noguchi Memorial Institute for Medical Research, University of Ghana, Legon, Accra, Ghana, ug.edu.gh; ^6^ Department of Internal Medicine, Section on Infectious Diseases, Wake Forest University School of Medicine, Winston-Salem, North Carolina, USA, wakehealth.edu; ^7^ Center for the Study of Microbial Ecology and Emerging Diseases, Wake Forest University School of Medicine, Winston-Salem, North Carolina, USA, wakehealth.edu; ^8^ Department of Biology, Wake Forest University, Winston-Salem, North Carolina, USA, wfu.edu

**Keywords:** dogs, *Ehrlichia canis*, Ghana, *Rhipicephalus sanguineus* s.l., *Rickettsia africae*

## Abstract

Tick‐borne pathogens, transmitted by ticks, infect humans and animals worldwide. The brown dog tick, *Rhipicephalus sanguineus* sensu lato, is a significant vector of a number of pathogens, including *Ehrlichia canis*, *Rickettsia* and *Anaplasma* species. In Ghana, there is limited information on the pathogens carried by *Rh. sanguineus* s.l. As such, *Rh. sanguineus* ticks taken from domestic dogs in Kumasi were screened for tick‐borne pathogens, including *Coxiella burnetii*, *Rickettsia*, *Babesia*, *Theileria*, *Anaplasma*, *Ehrlichia* and *Hepatozoon* species. A total of 204 ticks collected from 56 infested dogs were morphologically identified as *Rh. sanguineus* s.l. From the 88 pools screened, 36 (40.9%) were positive for pathogen DNA. The pathogens identified were *Rickettsia africae* (5 pools), *Ehrlichia canis* (10 pools) and uncultured *Anaplasma* sp. (21 pools) with maximum likelihood estimates as 2.48% (95% CI: 0.93, 5.38%), 5.22% (95% CI: 2.69, 9.15%) and 11.20% (95% CI: 7.32, 16.29%), respectively. There was no association between the detection of a pathogen and the tick sex or dog breed, age or sex. This study provides important baseline data on the circulation of tick‐borne pathogens in *Rh. sanguineus* s.l. ticks in Kumasi, with implications for both veterinary and human health. The presence of uncultured *Anaplasma* sp. suggests a wider diversity of tick‐borne bacteria with unknown pathogenicity. There is a need for integrated tick control, improved diagnosis and additional epidemiological studies to mitigate the impact of tick‐borne diseases in Ghana.

## 1. Introduction

Ticks are obligatory hematophagous vectors that transmit numerous pathogens that negatively impact people and animals worldwide [1]. Due to its widespread distribution and intimate relationship with domestic dogs, which are common companion animals in both urban and rural areas, *Rhipicephalus sanguineus* s.l., often known as the brown dog tick, is of special veterinary and public health significance [2]. Numerous pathogens that cause substantial morbidity and mortality in domestic animals and, occasionally, in humans are spread by *Rh. sanguineus* s.l. [2, 3]. A typical example is *Ehrlichia canis,* which causes canine ehrlichiosis in dogs [4]. Other pathogens, such as *Anaplasma* and *Rickettsia* species, have also been reported in *Rh. sanguineus* s.l. ticks collected from dogs [5]. In Ghana, dogs in Kumasi have been reported as infected with pathogens including *Ehrlichia canis*, *Hepatozoon canis* and *Anaplasma platys* [6]. Furthermore, *Rh. sanguineus* s.l. collected from dogs were found infected with *Rickettsia* spp. [7]. These findings indicate the importance of dog ticks and the need to include them in surveillance efforts to prevent infection spread. The second‐largest city in Ghana, Kumasi, is known for its high human population and close contact with domestic animals, especially dogs. Humans, domestic animals and ticks frequently interact in the urban and peri‐urban environment, which makes it perfect for the spread of diseases carried by ticks. Despite this, little is known about the variety and frequency of tick‐borne pathogens in *Rh. sanguineus* s.l. ticks in dogs in this area. Knowing the distribution and abundance of these tick‐borne pathogens is essential for determining the risk of infection for humans and dogs and developing effective control measures.

This study was therefore carried out to molecularly screen *Rh. sanguineus* ticks obtained from domestic dogs in Kumasi, Ghana, for the presence of tick‐borne pathogens of zoonotic and veterinary importance. The results add to the expanding body of information on West African tick‐borne diseases and draw attention to the possible veterinary and public health consequences of infections in an urban African environment.

## 2. Methods

In this cross‐sectional study, ticks were collected from 56 infested domestic dogs from April to May 2025 at two locations in urban Kumasi: Catena Veterinary Clinic (6.67687, −1.61158) and Adagya (6.62026, −1.57909). At Adagya, tick collection was carried out during a community dog vaccination programme, where ticks were removed from visibly infested dogs. Each dog was physically examined by a veterinarian, and ticks were removed using blunt forceps and stored in 15 mL Falcon tubes containing 70% ethanol. Information such as location, dog breed, age and sex was recorded for each infested dog. In the laboratory, the ticks were identified morphologically using available taxonomic keys [8] and pooled (1–6) based on the sex of the tick species. Total nucleic acid was extracted from the pools using the QIAamp Extraction Kit (Qiagen, Valencia, CA, USA) according to the manufacturer’s instructions [9].

Ethical clearance for this study was obtained from the Animal Research Ethics Committee (AREC) of the Kwame Nkrumah University of Science and Technology (KNUST 0087).

### 2.1. Pathogen Detection

The samples were screened for *Coxiella burnetii* by the amplification of 687 bp using an assay targeting the IS1111 fragment, a transposon‐like repetitive region [10, 11]. Primers that target the *Rickettsia* rOmpA gene (ompA) were used to perform a 632 bp amplification to detect *Rickettsia* DNA in ticks [12]. The samples were also screened for *Babesia*/*Theileria* DNA using primers that target the 560 bp portion of the *Babesia* and *Theileria* ssrRNA gene [13]. Primers targeting the 345 bp region of the *Ehrlichia* genus 16S rRNA gene were used to detect the presence of *Ehrlichia*/*Anaplasma* DNA in the ticks [14]. An assay that amplifies the 18S rRNA gene of *Hepatozoon* species at 666 bp was used to screen the ticks for *Hepatozoon* species [15].

The positive PCR products were subsequently purified and sequenced using Sanger sequencing.

### 2.2. Phylogenetic and Statistical Analysis

Following a review of the nucleotide sequences produced in this investigation using Chromas (Version 2.6.6), a consensus sequence was edited, cleaned and created using MEGA (Version 12.0.11). Next, each sequence was contrasted with other sequences (https://blast.ncbi.nlm.nih.gov/Blast.cgi) in the NCBI database. MEGA’s Clustal Omega programme was used for sequence alignment, and the maximum likelihood approach was used to build phylogenetic trees [16]. A thousand bootstrap replicates were used to calculate the confidence indices inside the phylogenetic trees, and the findings were shown as percentages on the branches. For the GenBank sequences used in the phylogenetic analysis, the different accession numbers and countries of origin have been specified. We used descriptive statistics, such as counts and proportions, to summarise the data. Next, we determined each pathogen’s minimum infection rate, providing the precise 95% CIs as well as the point estimate. We also used the PooledInfRate tool (https://github.com/CDCgov/PooledInfRate) to compute the pooled prevalence estimate. Tick species and tick species within each location were used to stratify these estimations. Next, we used Poisson regression and clustered errors to evaluate those characteristics linked to higher prevalence ratios (PRs) for each detected pathogen. We adjusted for animal identification and pool size using an offset. R Version 4.3.3 was used for all analysis.

## 3. Results

A total of 56 dogs were examined from Catena Veterinary Clinic (25) and Adagya (31) (Table 1). Most of the dogs were mongrels (71%), male (61%) and had a median age of 8 months. All the 204 adult ticks collected from the dogs (average of 3.64 ticks per dog) were identified as *Rhipicephalus sanguineus* s.l. The males were 109 (53.43%), while the females were 95 (46.57%).

**Table TABLE 1:** Characteristics of the infested dogs and sampled ticks.

Characteristics	*N* = 56
Location, *n* (%)	
Adagya	31 (55)
Catena Veterinary Clinic	25 (45)
Dog breed, *n* (%)	
Boerboel	1 (1.8)
Cane Corso	2 (3.6)
Caucasian Shepherd	1 (1.8)
French Bulldog	1 (1.8)
German Shepherd	2 (3.6)
Husky	1 (1.8)
Maltese	3 (5.4)
Mongrel	40 (71)
Pitbull	1 (1.8)
Poodle	1 (1.8)
Rottweiler	3 (5.4)
Dog sex, *n* (%)	
Female	22 (39)
Male	34 (61)
Dog age (months), median (IQR)	8 (4–24)

From the 88 pools screened, pathogen DNA was detected in 36 (40.9%) tick pools. The pathogens identified were *R. africae* in 5 (5.68%) tick pools, *Ehrlichia canis* in 10 (11.36%) pools and uncultured *Anaplasma* sp. in 21 (23.86%) pools. Generally, the MLE of the identified pathogens was 2.48% (95% CI: 0.93, 5.38%), 5.22% (95% CI: 2.69, 9.15%) and 11.20% (95% CI: 7.32, 16.29%) for *R*. *africae*, *E*. *canis* and uncultured *Anaplasma* sp., respectively (Table 2). The MLE of uncultured *Anaplasma* sp. in the tick pools was 11.08% (95% CI: 5.32, 20.07%) from Adagya and 11.19% (95% CI: 6.51, 17.81%) from Catena Veterinary Clinic (Table 3). The MLE of the zoonotic pathogen *R. africae* was 6.67% (95% CI: 2.53, 14.14%) in tick pools obtained from Adagya. There was no association between the detection of a pathogen and the tick sex or dog breed, age or sex (Table 4). None of the tick pools were positive for *C. burnetii*, *Babesia*, *Theileria* or *Hepatozoon* DNA.

**Table TABLE 2:** General infection rates for the sampled ticks.

Species	Pathogen	Positives (*n*/*N* pools)	Pool infection rate (%)	Minimum infection rate (%)	Maximum likelihood
*Rh. sanguineus* s.l.	*E*. *canis*	10/88	11.36 (5.59–19.91)	4.93 (2.39–8.87)	5.22 (2.69–9.15)
*Rh. sanguineus s*.l.	*R*. *africae*	5/88	5.68 (1.87–12.76)	2.46 (0.80–5.65)	2.48 (0.93–5.38)
*Rh. sanguineus* s.l.	Uncultured *Anaplasma* sp.	21/88	23.86 (15.42–34.14)	10.34 (6.52–15.38)	11.20 (7.32–16.29)

**Table TABLE 3:** Infection rate for ticks collected from dogs in Kumasi by location.

Location	Species	Pathogen	Positives (*n*/*N* pools)	Pool infection rate (%)	Minimum infection rate (%)	Maximum likelihood
Adagya	*Rh. sanguineus* s.l.	*Ehrlichia canis*	2/42	4.76 (0.58–16.16)	2.60 (0.32–9.07)	2.61 (0.47–8.29)
Adagya	*Rh. sanguineus* s.l.	*Rickettsia africae*	5/42	11.90 (3.98–25.63)	6.49 (2.14–14.51)	6.67 (2.53–14.14)
Adagya	*Rh. sanguineus* s.l.	Uncultured *Anaplasma* sp.	8/42	19.05 (8.60–34.12)	10.39 (4.59–19.45)	11.08 (5.32–20.07)
Catena Veterinary Clinic	*Rh. sanguineus* s.l.	*Ehrlichia canis*	8/46	17.39 (7.82–31.42)	6.35 (2.78–12.13)	6.92 (3.29–12.81)
Catena Veterinary Clinic	*Rh. sanguineus* s.l.	*Rickettsia africae*	0/46	0.00 (0.00–7.71)	0.00 (0.00–2.89)	0.00 (0.00–2.96)
Catena Veterinary Clinic	*Rh. sanguineus* s.l.	Uncultured *Anaplasma* sp.	13/46	28.26 (15.99–43.46)	10.32 (5.61–17.00)	11.19 (6.51–17.81)

**Table TABLE 4:** Prevalence ratios associated with detection by pathogen.

Characteristics	*Ehrlichia canis*			*Rickettsia africae*			Uncultured *Anaplasma* sp.		
	N	IRR (95% CI)^1^	*p* value	N	IRR (95% CI)^1^	*p* value	N	IRR (95% CI)^1^	*p* value
Breed (dog)	88			88			88		
Mongrel		—			—			—	
Other		2.63 (0.68–10.2)	0.16		0.00 (0.00 to Inf)	> 0.99		1.07 (0.44–2.59)	0.88
Sex (dog)	88			88			88		
Female		—			—			—	
Male		0.49 (0.12–1.95)	0.31		157, 143, 015, 689, 921 (0.00 to Inf)	0.97		0.98 (0.41–2.33)	0.96
Age (months)	88	1.00 (0.94–1.06)	0.97	88	0.97 (0.89–1.05)	0.48	88	1.02 (0.98–1.05)	0.36
Sex (tick)	88			88			88		
Female		—			—			—	
Male		3, 119, 900, 944, 862, 863 (0.00 to Inf)	0.94		0.00 (0.00 to Inf)	> 0.99		1.40 (0.58–3.38)	0.45

^1^IRR = incidence rate ratio; CI = confidence interval.

### 3.1. Basic Local Alignment Search Tool and Phylogenetic Analysis

It was observed that the sequences E15 and E16 were 100% similar to uncultured *Anaplasma* sp. from Palestine (MK069488). The sequence E17 was also 100% similar to *E. canis* from Brazil (MZ323324), and the sequence R10 was 98% similar to *R. africae* from Benin (KT633264). From the phylogenetic tree, it was seen that the sequences E15 and E16 were related to uncultured *Anaplasma* sp. reported from Palestine and the Philippines, while the sequence E17 was related to *E. canis* from Brazil (Figure 1). Sequence R10 was found to be closely related to *R. africae* reported from Ghana and Benin (Figure 2).

**Figure FIGURE 1:**
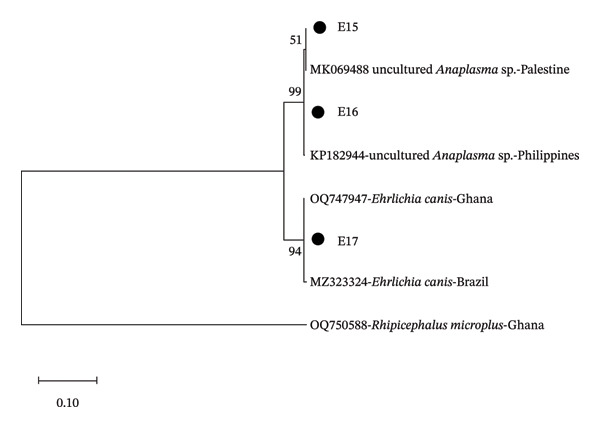
Phylogenetic analysis of the identified *Ehrlichia* and *Anaplasma* based on the 16S rRNA gene. The sequences from this study are E15, E16 and E17.

**Figure FIGURE 2:**
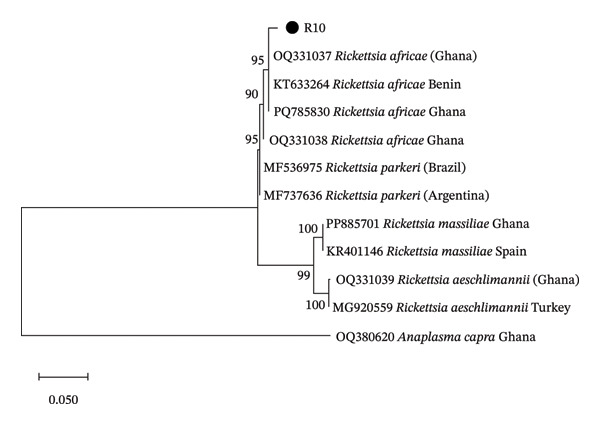
Phylogenetic analysis of the identified *Rickettsia* based on the OmpA gene. The sequence ID from this study is designated as R10.

The sequences generated in this study have been submitted to GenBank with accession numbers: uncultured *Anaplasma* sp. (PV861966 and PV861967), *E. canis* (PV861968) and *R. africae* (PV870140).

## 4. Discussion

*Rhipicephalus sanguineus* s.l. ticks collected from domestic dogs in Kumasi were infected with tick‐borne pathogens of zoonotic and veterinary importance.

*Ehrlichia canis* was identified in 11.36% of the tick pools in this study. This finding is higher than the *E*. *canis* prevalence of 6.7% reported previously in Ghana [17] but lower than 23.7% reported in a study from Nigeria [5]. This pathogen causes the disease canine ehrlichiosis, a common and frequently underdiagnosed disease in tropical Africa that affects dogs [4, 18]. Its clinical symptoms can range from mild to severe and include fever, anaemia, bleeding abnormalities and immunosuppression [18]. The fact that *E. canis* was found in the sampled ticks suggests that domestic dogs are involved in transmission cycles and could act as infection reservoirs. Reports of seropositivity in humans exposed to *Rh. sanguineus* s.l. ticks and reported instances in immunocompromised persons and blood donors underscore a possible zoonotic danger, notwithstanding the rarity of human ehrlichiosis caused by *E. canis* [19–22]. This emphasises how crucial it is for veterinarians and public health professionals in Kumasi to be aware of integrated tick management strategies.

In this study, uncultured *Anaplasma* sp. was also detected in the *Rh. sanguineus* s.l. ticks. Their intracellular nature and the absence of culture methods make it difficult to characterise these uncultured strains [23, 24], but their identification in ticks that parasitise domestic dogs raises questions regarding their potential to be pathogenic. Thrombocytopenia, lethargy and fever are common symptoms of *Anaplasma* infections in dogs, which can make veterinary diagnosis and treatment more difficult [25, 26]. Furthermore, caution is required due to the zoonotic potential of some *Anaplasma* species [23], particularly in areas where tick exposure is common. To determine the role of the identified uncultured *Anaplasma* sp. in animal and human disease, additional molecular characterisation and epidemiological research are necessary.

This study reports the detection of *R*. *africae* in *Rh. sanguineus* s.l. The detection of *R. africae* in 5.68% of the tick pools from this study is higher than the 2.2% reported by a previous study in Ghana [17]. *Rickettsia africae*, the causative agent of African tick‐bite fever (ATBF), is primarily transmitted by *Amblyomma variegatum* and *Amblyomma hebraeum* [27]. However, *Rhipicephalus* and *Hyalomma* ticks that cofeed with *Amblyomma* ticks can be infected with *R. africae* [28]. Among travellers and rural people in sub‐Saharan Africa, ATBF is a major cause of febrile illness [29]. Studies in Ghana focussed on livestock ticks have reported the occurrence of *R. africae* [30–33]. With the frequent interactions among domestic animals in Ghana, there is the risk of tick or tick‐borne pathogen exchange [34]. Given the presence of *R. africae* in ticks that infest domestic dogs, dogs may serve as amplifying hosts or reservoirs, which could raise the danger of human exposure in urban settings. Since *Rh. sanguineus* frequently infests dogs in homes, there is a chance that humans could become infected via tick bites, particularly in areas where people are not well informed or take precautions against tick bites [2]. Increased surveillance, prevention of tick bites and the inclusion of *R. africae* infection in the differential diagnosis of feverish diseases in the region are all recommended by this finding.

The complicated epidemiology of tick‐borne illnesses in urban African environments is highlighted by the codetection of all three pathogens in *Rh. sanguineus* ticks from Kumasi. Domestic dogs facilitate the maintenance and spread of several tick‐borne diseases in humans by acting as significant hosts and reservoirs [35]. Tick bites and associated pathogen transmission are more likely in Kumasi due to the proximity of people and dogs. The findings of this study bring out two challenges: effectively diagnosing and managing tick‐borne infections in dogs to better protect animal health and reducing the risk of zoonotic infections to safeguard human health. Regular tick control for dogs, public education campaigns on preventing tick bites and enhanced diagnostic tools for tick‐borne illnesses in the veterinary and human healthcare sectors are all examples of effective control measures. Furthermore, continuous molecular surveillance will be an effective method for detecting circulating pathogens and monitoring the risk of emerging threats.

## 5. Conclusion

This study reports the occurrence of *E. canis*, *R. africae* and uncultured *Anaplasma* sp. in *Rh. sanguineus* s.l. collected from domestic dogs. The identification of these pathogens in tick species that are closely linked to humans and dogs highlights the possible danger of tick‐borne diseases in veterinary and public health settings. There is an urgent need for integrated surveillance and control strategies to reduce the risk posed to the health of both domestic animals and humans.

## Funding

No funding was received for this manuscript.

## Conflicts of Interest

The authors declare no conflicts of interest.

## Data Availability

All the data supporting this study are included in the article.
